# Balancing Human Mobility and Health Care Coverage in Sentinel Surveillance of Brazilian Indigenous Areas: Mathematical Optimization Approach

**DOI:** 10.2196/69048

**Published:** 2025-04-01

**Authors:** Juliane Fonseca Oliveira, Adriano O Vasconcelos, Andrêza L Alencar, Maria Célia S L Cunha, Izabel Marcilio, Manoel Barral-Netto, Pablo Ivan P Ramos

**Affiliations:** 1 Center for Data and Knowledge Integration for Health, Gonçalo Moniz Institute, Fundação Oswaldo Cruz, Parque Tecnológico da Edf. Tecnocentro, R. Mundo, 121 - sala 315 - Trobogy, Salvador, 41745-715, Brazil, 55 71 3176 2357; 2 Luiz Coimbra Institute of Graduate and Engineering Research, Federal University of Rio de Janeiro, Rio de Janeiro, Brazil; 3 Department of Computer Science, Federal Rural University of Pernambuco, Recife, Brazil; 4 Bahiana School of Medicine and Public Health (EBMSP), Salvador, Brazil; 5 Medicine and Precision Public Health Laboratory, Gonçalo Moniz Institute, Fundação Oswaldo Cruz, Salvador, Brazil

**Keywords:** representative sentinel surveillance, early pathogen detection, indigenous health, human mobility, surveillance network optimization, infectious disease surveillance, public health strategy, Brazil

## Abstract

**Background:**

Optimizing sentinel surveillance site allocation for early pathogen detection remains a challenge, particularly in ensuring coverage of vulnerable and underserved populations.

**Objective:**

This study evaluates the current respiratory pathogen surveillance network in Brazil and proposes an optimized sentinel site distribution that balances Indigenous population coverage and national human mobility patterns.

**Methods:**

We compiled Indigenous Special Health District (Portuguese: Distrito Sanitário Especial Indígena [DSEI]) locations from the Brazilian Ministry of Health and estimated national mobility routes by using the Ford-Fulkerson algorithm, incorporating air, road, and water transportation data. To optimize sentinel site selection, we implemented a linear optimization algorithm that maximizes (1) Indigenous region representation and (2) human mobility coverage. We validated our approach by comparing results with Brazil’s current influenza sentinel network and analyzing the health attraction index from the Brazilian Institute of Geography and Statistics to assess the feasibility and potential benefits of our optimized surveillance network.

**Results:**

The current Brazilian network includes 199 municipalities, representing 3.6% (199/5570) of the country’s cities. The optimized sentinel site design, while keeping the same number of municipalities, ensures 100% coverage of all 34 DSEI regions while rearranging 108 (54.3%) of the 199 cities from the existing flu sentinel system. This would result in a more representative sentinel network, addressing gaps in 9 of 34 previously uncovered DSEI regions, which span 750,515 km² and have a population of 1.11 million. Mobility coverage would improve by 16.8 percentage points, from 52.4% (4,598,416 paths out of 8,780,046 total paths) to 69.2% (6,078,747 paths out of 8,780,046 total paths). Additionally, all newly selected cities serve as hubs for medium- or high-complexity health care, ensuring feasibility for pathogen surveillance.

**Conclusions:**

The proposed framework optimizes sentinel site allocation to enhance disease surveillance and early detection. By maximizing DSEI coverage and integrating human mobility patterns, this approach provides a more effective and equitable surveillance network, which would particularly benefit underserved Indigenous regions.

## Introduction

Sentinel surveillance, which involves systematic and regular clinical sample collection for monitoring the emergence of infectious diseases across a network of sites, is a key component of public health strategies [1]. Ideally, a sentinel surveillance network should be representative of the general population while also prioritizing high-risk subpopulations, such as Indigenous groups [2-5].

Major global sentinel networks include the Global Influenza Surveillance and Response System established by the World Health Organization, which operates across 127 countries [6]; the European Influenza Surveillance Network [7], which relies on reports from general practitioners to monitor flu activity; and SARInet plus (Severe Acute Respiratory Infections Network) in the Americas, which is coordinated by the Pan American Health Organization [8]. These networks are essential for monitoring flu cases, detecting emerging respiratory viruses, guiding public health interventions and vaccine development, and standardizing global sentinel surveillance practices.

The effectiveness of and capability for early pathogen detection depend on local routine surveillance, which varies widely across regions and faces significant challenges [6,9]. In fact, despite the falling costs of genomic sequencing and advancements in metagenomics allowing for the monitoring of multiple pathogens and the identification of novel agents, low- and middle-income countries still struggle to scale up sentinel surveillance due to financial constraints, as well as a lack of political support for and investment in technological and scientific developments [10-13]. As a result, sentinel sites are often chosen based on convenience, leaving populations at high risk and important target regions, such as Indigenous communities, underserved. In this context, the use of alternative data streams to understand pathogen emergence and the likely pathways of disease spread may maximize the available information for improving the data-driven allocation of surveillance resources [11,13-16]. In particular, this approach enables the incorporation of epidemiological intelligence into planning the target population and geographical location for sentinel clinical sample collection. Motivated by this, we integrated human mobility data with Indigenous population coverage to refine sentinel site selection in Brazil.

The boundaries between wild landscapes and human settlements have been increasingly blurred by climate change, agricultural and urban expansion, deforestation, and landscape fragmentation. These compounding factors heighten Indigenous communities’ vulnerability to infectious disease spillover, which can rapidly spread to urban areas [17]. In addition, Brazilian Indigenous populations face significant barriers to health care access, limiting their awareness of circulating diseases and disproportionately increasing their disease burden [2-4,18]. During the COVID-19 pandemic, the incidence of and mortality resulting from the disease among Indigenous and traditional people were higher when compared to those among the general population, highlighting the need for tailored public health strategies to address health disparities in these communities [19-22].

We recently showed how human mobility patterns can inform the redesign of existing sentinel networks to improve early pathogen detection [13]. However, the high risk of pathogen emergence in Indigenous communities may remain undetected when relying solely on mobility data, potentially delaying the identification of health threats in these populations. To address this gap, we aimed to build and expand on our mobility-based model by explicitly incorporating the geographical distribution of Indigenous communities in Brazil. In particular, we aimed to provide an optimized list of cities that could serve as strategic candidate sites for early pathogen detection in Brazil, while also adding an equity component into sentinel planning.

## Methods

### Data Sources

For our analyses, we collected data on cities within Brazilian Indigenous Special Health Districts (Portuguese: Distrito Sanitário Especial Indígena [DSEI]), which were obtained from the Brazilian Ministry of Health, along with mobility coverage data derived from the Ford-Fulkerson algorithm [13,23].

To ensure the practical applicability of our results, we also collected information on the composition of Brazil’s current influenza sentinel surveillance network, which was obtained through direct communication with the Ministry of Health. We also incorporated a health attraction index, which quantifies a city’s potential to attract individuals seeking health care services. This index, as estimated by the Brazilian Institute of Geography and Statistics [24], categorizes cities based on their capacity to provide low-, medium-, and high-complexity health services, and its incorporation further refined our sentinel site selection process. The complete dataset is available in a GitHub directory [25].

### Optimization Problem

To identify the most suitable sentinel sites for maximizing both human mobility coverage and the representation of DSEIs, we applied an optimization approach.

Brazil’s 34 DSEIs are administrative regions designed to manage health activities for Indigenous territories in an ethnically and culturally sensitive manner. DSEIs encompass 1470 of Brazil’s 5570 municipalities, with partial overlap in 46 others. Overlaps occur because Indigenous territories often span multiple municipalities or states, and DSEIs are designed to align with Indigenous geography rather than political borders. In our work, we considered a DSEI to be covered in the sentinel network if at least 1 municipality completely located within a DSEI was included in the design of the network.

To define mobility coverage, we constructed an intercity mobility network, which was represented as graph G=(V, E), where the set of nodes (V) corresponds to cities, and the set of links (E) represents movement frequencies between them. Using this network, we applied the Ford-Fulkerson algorithm [13] to model potential disease spread pathways, identifying the most probable routes for pathogen transmission from a source city. This approach allowed us to determine key locations for early detection by analyzing how frequently cities appeared at different stages in these transmission paths. Further methodological details are available in our previously published paper [13]. Based on the output of the Ford-Fulkerson algorithm, we defined a city’s mobility coverage over another city, as follows: city A covers city B if n% of the most likely transmission paths originating from city B include city A as their first step; n is a value between 0 and 100, and when n=100, it is considered that city A fully covers city B.

To maximize mobility coverage while ensuring that all DSEI regions are represented, we formulated this as an optimization problem. Mathematically, we let *x_i_* be a binary variable, where *x_i_*=1 if city *i* is selected for the sentinel network and *x_i_*=0 otherwise. Further, we let *ω_i_* represent the mobility coverage weight of city *i*. Additionally, we let *R_j_* denote the set of cities within DSEI region *j* and let *N* be the total number of cities to be selected for the sentinel network. The optimization function was then defined as follows:


$$max\left(\sum_{i}^{\;}\;\omega_{i}x_{i}\right)$$

subject to

1. $\sum_{i}{\;}^{\;}x_{i}\;=\;N$, that is, select *N* cities to compose the sentinel.

2. $\sum_{j\in R_{j}}^{\;}x_{j}\geq \;1,$for all $j\in \left\{1,2,...,34\right\}$, that is, for each DSEI region *j*, at least 1 city in *R_j_* is selected.

The optimization problem was implemented in Python and solved by using the Python Universal Linear Programming library [25].

### Practical Applicability

Brazil’s flu sentinel network primarily requires sites in capital cities, with 1 site per 500,000 inhabitants, except in the south region, where 1 site is implemented for every 300,000 inhabitants, regardless of metropolitan status. Additionally, sample collection occurs in intensive care unit services that cover approximately 10% of the available intensive care unit beds in each municipality, ensuring coverage across age groups [26].

To assess the practical applicability of our proposed sentinel network, we compared it with Brazil’s existing flu sentinel network to evaluate improvements in mobility and Indigenous region coverage. We also assessed whether the newly identified cities serve as key access points for health care services across different levels of complexity. This evaluation reinforced that the proposed sentinel locations align with urban centers that attract individuals seeking health care, making them strategic candidates for genomic sample collection.

### Ethical Considerations

This study used publicly available secondary data sources. No personal data were collected or analyzed, ensuring the complete anonymity of all data. Ethical approval was not required, as the dataset consisted of anonymized, aggregated information, following resolutions 466/2012 and 510/2016 (article 1, sections III and V) from the National Health Council (Conselho Nacional de Saúde), Brazil. All analyses were carried out in accordance with relevant ethical guidelines for data protection and research integrity.

## Results

The current network includes 310 sentinel sites in 199 municipalities, representing only 3.6% (199/5570) of the country’s cities. To compose a more representative sentinel network for the country, we optimized the selection of 199 cities—the same number considered in Brazil’s current flu sentinel network. In the optimized network, we achieved 100% DSEI coverage by selecting the cities (87/199, 43.7%) that are fully inside of DSEI regions. In addition, 69.2% of the country’s mobility pattern is covered by these cities, translating to 6,078,747 paths (of 8,780,046 total paths) originating from each city in the country.

**Figure 1.**
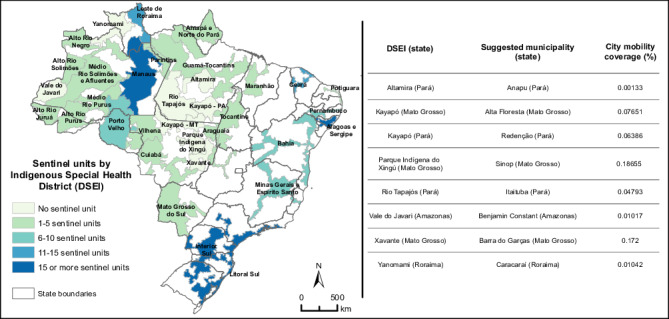
Left: distribution of active surveillance sites within DSEIs in Brazil (2025). This map illustrates the current coverage of DSEIs by the national influenza surveillance network, highlighting the geographical distribution of sentinel sites across Indigenous regions. Colored areas represent DSEI regions according to the number of sentinel units. Right: proposed optimized list of municipalities within underserved Indigenous regions not currently covered by the Brazilian flu sentinel network, along with their corresponding mobility coverage. This list, which was generated from the optimization process, aims to improve DSEI coverage while incorporating Brazil’s national human mobility patterns. The proposed cities guide resource allocation to enhance surveillance effectiveness in the country’s underserved Indigenous areas. DSEI: Distrito Sanitário Especial Indígena.

In the current network, 53.8% (107/199) of the municipalities overlap 25 DSEIs (Figure 1). However, the following nine DSEIs lack any sentinel coverage: Altamira (Pará), Kayapó (Mato Grosso), Kayapó (Pará), Médio Rio Purus (Amazonas), Parque Indígena do Xingu (Mato Grosso), Rio Tapajós (Pará), Vale do Javari (Amazonas), Xavante (Mato Grosso), and Yanomami (Roraima). These regions span more than 750,515 km² and are home to more than 1.11 million people. In addition, Brazil’s current flu sentinel network covers only 52.4% (4,598,416 of 8,780,046 paths) of mobility patterns (considering air, road, and waterway transportation modes).

By rearranging 108 cities in Brazil’s flu sentinel network—keeping 91 cities that are already included in the current network—we achieved 100% DSEI coverage and increased national mobility coverage by 16.8 percentage points (from 52.4% to 69.2%). Figure 1 shows the list of municipalities that would ensure coverage for DSEIs without sentinel sites in Brazil’s flu sentinel network.

Finally, we analyzed the movement of people seeking health care in the cities selected for the optimized sentinel network. None of the identified cities had a low health care attraction index. The majority (192/199, 96.5%) of cities serve as hubs for high-complexity health care services, while 3.5% (7/199) are classified as medium-complexity hubs. Among cities within DSEIs, 33% (29/87) have a high health care attraction index and are not currently covered by Brazil’s flu sentinel network. Additionally, 67% (58/87) have a medium attraction index, with 4 lacking sentinel coverage.

## Discussion

In this study, we present an optimized list of 199 cities that would enhance early pathogen detection by strategically balancing Indigenous area coverage and human mobility patterns. Our selection ensures 100% coverage of Indigenous regions through 87 key cities while also capturing 69.2% of the country’s human mobility flows. Additionally, the feasibility of implementing sentinel surveillance in these locations is supported by their high health care attraction index, which indicates their role as hubs for complex health care services.

Our findings introduce a novel approach that integrates human mobility and Indigenous population coverage to optimally rearrange Brazil’s current flu sentinel network locations for clinical sample collection. By focusing on the historically excluded Indigenous communities, the proposed approach aligns with expert recommendations to enhance surveillance in underserved areas [2-4,10], thereby ultimately protecting vulnerable communities while improving Brazil’s ability to detect and respond to emerging health threats.

By ensuring that all DSEIs are represented by at least 1 sentinel site, we addressed surveillance gaps in 9 previously uncovered regions that, collectively, are home to over 1 million people. This outcome is particularly significant, given the increased disease risks faced by Indigenous populations residing at the wildlife-urban interface. Our mobility-based optimization, which was combined with a restriction to 199 sentinel cities, reduced the number of cities within DSEIs when compared to Brazil’s current flu sentinel network. This highlights the following two key considerations: (1) some DSEI regions covered in the existing network may be overserved, and (2) the total number of cities selected for sample collection may be insufficient to account for Brazil’s geographic and epidemiological diversity, potentially limiting early pathogen detection. Nevertheless, maintaining or expanding routine sentinel surveillance is paramount to establishing a baseline of case incidence and tracking circulating viruses [1], which enable the early detection of outbreaks and the introduction of emergent pathogens. For instance, the absence of baseline testing likely contributed to the delay in the recognition of the Zika virus outbreak in the Americas, which peaked in 2015 to 2016 but probably spread undetected since late 2013 [27,28].

The optimized sentinel network achieved 69.2% mobility coverage—an improvement over Brazil’s current flu sentinel network. This shows that incorporating Indigenous population criteria resulted in the optimized network’s mobility coverage being only slightly lower than the 70% we reported [13] for a network based solely on mobility patterns. This underscores the need to examine the key epidemiological priorities (which may include socioeconomic deprivation and regions with a high risk of climate-related disasters) that should be taken into account when designing a representative sentinel network for early pathogen detection, as these factors may impact coverage metrics. Consequently, identifying an optimal balance between the minimum number of cities required for sentinel surveillance and the diversity of epidemiological priorities remains a critical area for future research.

Establishing a sentinel surveillance network, including a pipeline from sample collection to laboratory analysis, demands substantial resources, especially in regions with limited infrastructure. To address this challenge, we strategically centered our focus around DSEIs—an already in-place health administrative structure in Brazil that provides primary care to Indigenous populations and articulates with other networks in the Unified Health System to guarantee access to medium- and high-complexity services. Additionally, by evaluating the health attraction index and taking into account the outdated requirement for sentinel network implementation that was established by the Brazilian Ministry of Health [26], we found that the newly selected cities already offer medium- to high-complexity health care services—an indication that may support genomic sample collection. Leveraging this existing infrastructure not only facilitates routine surveillance but also strengthens overall health care capacity.

This work contributes novel insights to the literature by addressing the need for more effective selection of epidemiologically relevant criteria for early pathogen detection, while promoting a more equitable distribution of sentinel surveillance sites. To our knowledge, no previous study has explicitly optimized both underserved community coverage and human mobility patterns within a sentinel network framework. Existing research may only indicate standard criteria, such as gender, ethnicity, age, population coverage, and incidence of a specific disease [1,5,10], or rely on a single data source with anticipatory potential [29], without assessing whether these criteria are sufficient to truly maximize the chances of early threat detection. Our approach bridges this gap by offering a data-driven framework to enhance public health preparedness, and our findings may encourage the scientific community to discuss more about the importance of setting up and optimizing sentinel surveillance.

Our work has some limitations. Despite the significance of our findings, the data used in this study primarily identify the geographical regions encompassing Indigenous populations, without capturing other factors, such as climate-related risks or socioeconomic disparities, that may worsen these communities’ vulnerability to emerging new or unknown pathogens. Further research is needed to refine surveillance strategies tailored to these populations. Additionally, as we previously highlighted [13], access to up-to-date mobility data remains a key challenge. Finally, we emphasize that this study represents a step toward more data-driven approaches in designing sentinel surveillance networks, demonstrating how different epidemiological criteria (exemplified by the two considered herein) can be integrated into the optimization process to enhance disease monitoring.

To conclude, our results indicate that our proposed framework can be used to effectively guide the optimization of sentinel site allocation, and the framework can be further validated for use during new emergencies or in the monitoring of flu syndrome in Brazil. Our results contribute to increasing the likelihood of early pathogen detection without requiring an expansion in the number of sentinel site locations that have already been put into place by the Brazilian Ministry of Health, thereby addressing the critical challenge of limited funding—a well-documented barrier to improving global epidemiological surveillance, especially in low- and middle-income countries.

## Abbreviations

DSEI: Distrito Sanitário Especial Indígena
SARInet plus: Severe Acute Respiratory Infections Network
